# Impact on increasing the bent angle in nickel dibenzoporphyrin(2.1.2.1) bows. Evaluation of structural and molecular features from computations

**DOI:** 10.1039/d5ra07496g

**Published:** 2025-10-31

**Authors:** Margot Paco-Chipana, Alvaro Muñoz-Castro

**Affiliations:** a Doctorado en Biología Computacional, Facultad de Ingeniería, Universidad San Sebastián Bellavista 7 Santiago Chile; b Facultad de Ingeniería, Universidad San Sebastián Bellavista 7 Santiago Chile alvaro.munozc@uss.cl

## Abstract

Non-planar porphyrin motifs enable particular characteristics relevant for a wide range of applications. We explored (through computations) the features of increasing the bend in the more flexible dibenzoporphyrin (2.1.2.1) core. We decreased the bridge length from the recently reported butadiyne-bridged dibenzoporphyrin(2.1.2.1), and its nickel counterpart. Our results highlighted the flexibility of the dibenzoporphyrin(2.1.2.1) core, which could bend from 122.4° to 103.2° involving a strain energy of <3.4 kcal mol^−1^, and of 9.1 kcal mol^−1^ from 116.0° to 97.3° upon incorporating the central nickel atom. For further bent angles, destabilization of the dibenzoporphyrin(2.1.2.1) core increased to 23.7 and 38.5 kcal mol^−1^, respectively. This structural control modified the HOMO–LUMO gap and optical absorption patterns, and highlighted an increase in the Lewis acid capabilities of the resulting π-hole site perpendicular to the N4 molecular plane, in comparison with the parent nickel porphyrin, evaluated *via* calculated fluoride ion affinity. In addition, the induced magnetic field had a deshielding region ascribed to N4- and NiN4-motifs, which was retained within the bent cavity. These observations indicate that controlled bending of N4 is a useful synthetic strategy to tune the capabilities of porphyrin- and metalloporphyrin-like species.

## Abstract 2

Non-planar porphyrin motifs enable particular characteristics relevant for a wide range of applications.

## Graphical abstract

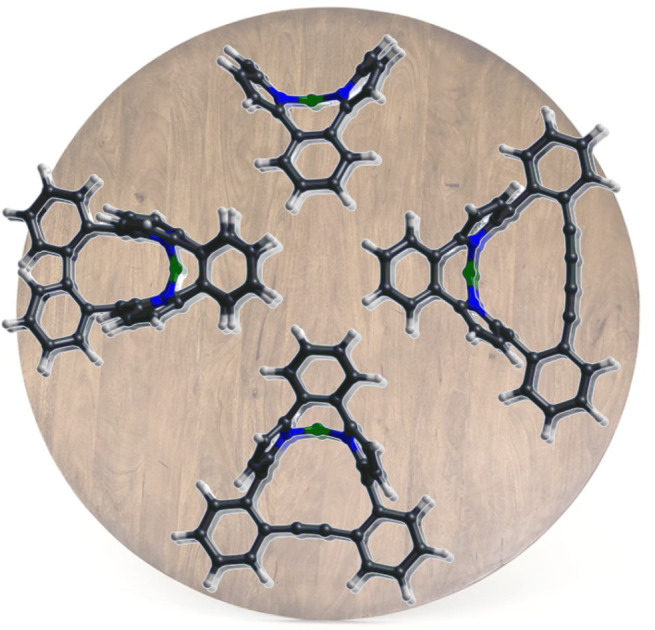

## Introduction

Non-planar porphyrin molecules exhibit intriguing features provided by their curved and strained structural backbones in comparison with planar motifs.^1–4^ These features lead to particular characteristics desired for applications ranging from biological models to functional electronic devices as sensors, optoelectronic, and photovoltaic species.^5–10^ The deviation from planarity can be induced and controlled by introducing the strain of the central motif, like in corannulene^11^ or tetraphenylene.^12,13^ This planarity deviation can be “tuned” by a delicate choice of the central structural core and the subsequent variation of peripheral substituents, among other approaches,^14–16^ resulting in a bowl- or saddle-shaped motif. The use of sterically demanding exo-substituents or cofacial aggregation^17,18^ has been employed to achieve an unprecedented perfectly planar corannulene structure. The use of a bridge connecting two different sites within the central core enables the introduction of a concave face in bent pyrenes.^19,20^

Early strategies to introduce non-planarity in porphyrin macrocyles have been developed by incorporating a bridging “bow limb” at the β-positions of the pyrrolic units,^21,22^ serving to stretch the planar porphyrin(1.1.1.1). Such an approach serves to increase the versatility of porphyrins and metalloporphyrins, resulting in interesting building blocks with tuned characteristics.^23–27^ Despite these achievements, the rigid characteristics of the porphyrin reduce the scope of planarity loss of the central structural core, where the use of inherently bent motifs serves to provide a more efficient curved situation.

In this respect, the work by Xue and coworkers explored the use of bent porphyrin structural cores given by the extended dibenzoporphyrin(2.1.2.1).^28–38^ This enabled an increase in the non-planarity of the coordinating N4-site,^39^ with interesting performance in electrocatalytic hydrogen evolution reaction (HER) activity for fused arrays. Furthermore, these authors conceived a mixed approach involving a bridging “bow limb” with the bent dibenzoporphyrin(2.1.2.1) core by using a butadiyne-bridge connected to exo-aryl rings within the dibenzoporphyrin(2.1.2.1) structure. This led to an increased strain, inducing a larger bowl-shaped deformation from planarity.^40^

We set out to explore the bent characteristics of the metal-free and nickel-incorporated butadiyne-bridged dibenzoporphyrin(2.1.2.1) (Por and PorNi, respectively),^40^ and further consequences of a more bent structure, by evaluating shorter-bridge situations involving acetylene- and directly-bridged exo-aryl rings. Our results are given in terms of the different energy contributions to the energy difference as the porphyrin bends, the resulting patterns of optical absorption, the induced magnetic field, and the variations at the N4-site. From such observations, further understanding of the capabilities in bending N4-motifs based on the bridged dibenzoporphyrin(2.1.2.1) core was evaluated, denoting the capabilities to tune different molecular characteristics.

## Computational details

Geometry optimizations and subsequent calculations were performed at the density functional theory (DFT) level employing the ADF code.^41,42^ We used the all-electron triple-ζ Slater basis set augmented with double polarization functions (STO-TZ2P) and the hybrid functional of Perdew, Burke, and Ernzerhof provided by Ernzerhof–Scuseria and Adamo–Barone (PBE0).^43–45^ London dispersion correction to DFT was taken into account by the pairwise method of Grimme (DFT-D3).^46^ Open-shell structures were treated under the unrestricted Kohn–Sham regime. Vibrational analysis was carried out to determine the minima character of the most favorable conformation, and the transition state nature of the involved transition states, as a first-order saddle point in the potential energy surface (one negative frequency). Time-dependent DFT (TD-DFT) calculations were carried out to simulate the optical UV-Vis spectrum for the studied series using all-electron TZ2P basis sets and the statistical averaging of different orbital-dependent model potentials (SAOP), which incorporate important features of the exact potential in the molecular and asymptotic regions, employed previously for metalloporphyrins and tetrapyrroles.^47^ To evaluate the magnetic response or induced field (**B**^ind^), upon an external magnetic field (**B**^ext^), a two-dimensional representation of the nucleus independent chemical shielding (NICS) tensor (*σ*_ij_) was obtained, where **B**_*i*_^ind^ = −*σ*_*ij*_**B**_*j*_^ext^.^48–52^ For convenience, the *i* and *j* suffixes correspond to the *x*-, *y*-, and *z*-axes of the molecule-fixed Cartesian system (*i*,*j* = *x*,*y*,*z*). **B**^ind^ is given in ppm in relation to **B**^ext^.

## Results and discussion

**Scheme 1:**
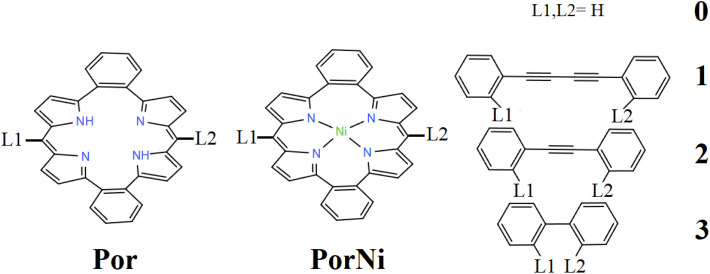
Por and PorNi cores, linked by L1 and L2 connection points for the giver bridges for 1, 2 and 3, and by H in 0.

**Fig. 1:**
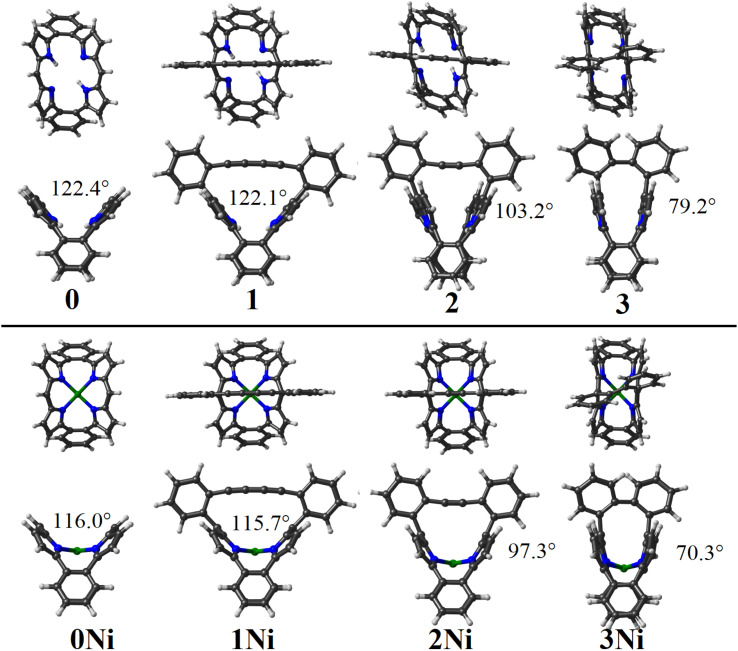
Calculated structures for the studied series, denoting the bent angle of the dibenzoporphyrin(2.1.2.1) motif, given from upper and side views.

The metal-free and nickel butadiyne-bridged dibenzoporphyrin(2.1.2.1), noted by Por and PorNi, respectively, are schematically given in Scheme 1. The optimized structures are given in Fig. 1, which denotes calculated distances between *ipso*-carbon from *meso*-phenyl units of 7.588 and 7.473 Å, which are in good agreement with the structurally characterized distances of 7.54 and 7.46 Å.^40^ For the free base, Por, the experimental distance from N and N–H to a central centroid was 2.007 and 2.179 Å, which has been calculated to be 1.980 and 2.177 Å.^40^ Upon inclusion of the Ni metal center in PorNi, experimental N–Ni distances amounted to 1.892 and 1.903 Å,^40^ which was calculated at 1.901 and 1.908 Å, respectively.

The angle between both *meso*-carbons and the N4-centroid was 123.5° and 116.2° for Por and PorNi, respectively, which was calculated to be 122.1° and 116.0°, showing a more bent structure upon inclusion of the Ni metal center. For the sake of simplicity, hereafter the Por and PorNi species are denoted as 1 and 1Ni.

We wished to evaluate the energy related to the induced deformation of the dibenzoporphyrin(2.1.2.1) core. We provided the energy difference between the unsubstituted dibenzoporphyrin(2.1.2.1) (0 and 0Ni) and its resulting structure within the 1 and 1Ni species. To offer a direct comparison, the bridging biphenyl butadiyne (-Ph–CCCC–Ph-), acetylene (-Ph–CC–Ph-) and biphenyl (-Ph-Ph-) motifs were removed and replaced by –H, for 1/1Ni, 2/2Ni and 3/3Ni, respectively. This enabled retention of their structures obtained in the overall structures (1/1Ni, 2/2Ni and 3/3Ni), thereby leaving the distorted dibenzoporphyrin(2.1.2.1) for a direct comparison. Results indicated a deformation energy of 0.9 and 2.0 kcal mol^−1^ upon inclusion of the butadiyne-bridged structure, which was bent to achieve its structure in 1 and 1Ni. Such values exposed the flexibility of the dibenzoporphyrin(2.1.2.1) core, which required a small amount of energy to increase its bowl-shape structure as given by the angle between both *meso*-carbons and the N4-centroid.

Furthermore, by replacing the butadiyne-bridge with an acetylene-based bridge, denoted by 2 and 2Ni, the distances between *ipso*-carbons from the *meso*-phenyl units in the free base (2) decreased to 5.801 and 5.817 Å in the nickel-incorporated counterpart (2Ni). This led to a narrower *meso*-carbons and N4-centroid angle of 103.2° and 97.3°, for 2 and 2Ni, respectively. This denoted a larger planarity deviation for the dibenzoporphyrin(2.1.2.1) core, which interestingly involved only a small destabilization of 3.4 kcal mol^−1^ for the freebase (2), which increased to 9.1 kcal mol^−1^ after Ni-coordination in 2Ni. This denoted that the metal-coordinated porphyrin core introduced a larger strain or rigidity for bent processes, serving to control the degree of deformation for the porphyrin core.

Removal of the acetylene-bridge, leaving both *meso*-phenyl rings to be directly connected, similar to a *para*-bisphenyl motif, resulted in shorter distances between *ipso*-carbons from the *meso*-phenyl units of 3.584 and 3.618 Å, for 3 and 3Ni, resulting in a very narrow angle between *meso*-carbons and the N4-centroid of 79.2° and 70.3°. Such an exaggerated induced bending of the dibenzoporphyrin(2.1.2.1) core involved deformation energies of 23.7 and 38.5 kcal mol^−1^, indicating that such structures were more destabilized compared with the above-discussed scenarios (1, 1Ni, 2, and 2Ni), making them challenging synthetic targets.

**Fig. 2:**
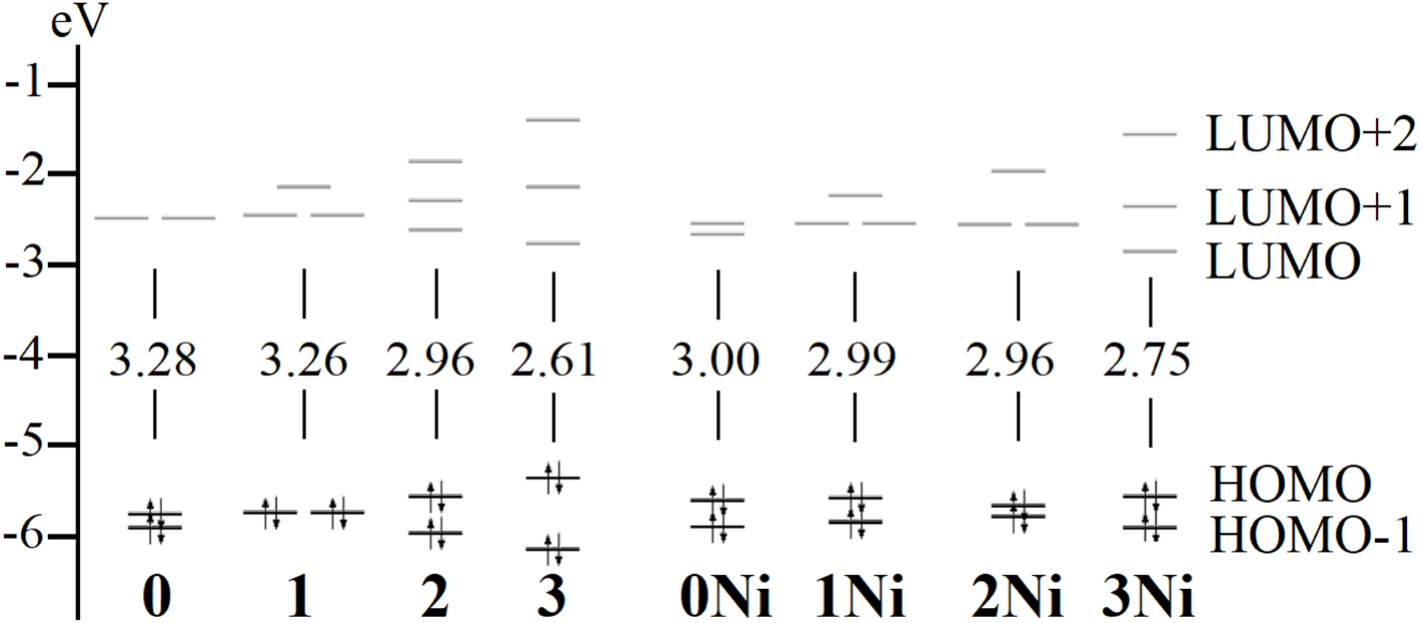
Calculated frontier orbitals and the respective HOMO–LUMO gap.

The calculated gap between frontier orbitals (HOMO–LUMO gap) ranged from 3.28 to 2.61 eV in decreasing 0 > 1 > 2 > 3 order (Fig. 2), denoting the role of reducing the angle between *meso*-carbons and N4-centroid angle, in frontier orbitals. For the nickel counterparts, the variation in the HOMO–LUMO gap was smaller, ranging from 3.00 to 2.75 eV, and decreased in the order 0Ni > 1Ni > 2Ni > 3Ni. Hence, the inclusion of the metal center hampered stronger variations in the frontier orbital gap. In the series, HOMO−1 and HOMO were of π-dibenzoporphyrin(2.1.2.1) character (Fig. S1); LUMO and LUMO+1 were of π*-dibenzoporphyrin(2.1.2.1) character. For the bridged species, LUMO+2 remained close to the frontier orbitals formed by bridge-based orbitals, which were destabilized from 1 to 3, and from 1Ni to 3Ni.

We wished to evaluate the variation of the N4–Ni coordination from 0Ni to 3Ni. The interaction energy (Δ*E*_int_) leading to the Ni coordination into the N4 cavity was calculated in terms of the Ni^2+^/ligand^2−^ fragmentation within the energy decomposition analysis.^53–55^ This resulted in a sizable stabilization of −906.1 kcal mol^−1^ for 0Ni, which decreased to −901.4, −897.7, and −887.6 for 1Ni, 2Ni and 3Ni, respectively (Table 1). Thus, despite the increasing deviation from planarity, the N4-cavity from the dibenzoporphyrin(2.1.2.1) core could retain a sizable Ni-coordination, which decreased stepwise according to the bent angle from 116.2° to 70.3°, with a destabilizing energy difference of 18.4 kcal mol^−1^ between 0Ni and 3Ni. The respective calculated bond dissociation energy (BDE) followed a similar trend to the discussed interaction energy, denoting a decrease from 0Ni to 3Ni (Table 1).

**Table 1:** Energy decomposition analysis for the Ni^2+^/ligand^2−^ fragmentation for the 0Ni to 3Ni series. In addition, related bond dissociation energies (BDEs) are provided. Values are in kcal mol^−1^

	0Ni		1Ni		2Ni		3Ni	
Δ*E*_Pauli_	289.7		285.3		294.42		301.04	
Δ*E*_elstat_	−668.2	55.9%	−652.6	55.0%	−653.8	54.8%	−649.6	54.6%
Δ*E*_orb_	−521.7	43.6%	−527.8	44.5%	−532.1	44.6%	−533.0	44.8%
Δ*E*_disp_	−5.8	0.5%	−6.3	0.5%	−6.2	0.5%	−6.1	0.5%
Δ*E*_int_	−906.1		−901.4		−897.7		−887.6	
BDE	850.2		846.3		835.5		815.2	

Moreover, within the framework of energy decomposition analysis^53–55^ provided by the Ziegler–Rauk energy decomposition scheme,^56^ the interaction energy (Δ*E*_int_) can be decomposed in several meaningful terms,^53–55^ given by:Δ*E*_int_ = Δ*E*_Pauli_ + Δ*E*_elstat_ + Δ*E*_orb_ + Δ*E*_disp_In this sense, the decrease in the interaction energy in the N4–Ni coordination from 0Ni to 3Ni involved contributions from the destabilizing Pauli repulsion accounting for the steric hindrance effects (Δ*E*_Pauli_), the variation in the stabilizing electrostatic character of the interaction (Δ*E*_elstat_), and the stabilizing orbital character (Δ*E*_orb_).^42^ In addition, the contribution from London interactions (Δ*E*_disp_) was accounted for by the pairwise correction from Grimme (D3).^57,58^

Interestingly, the stabilizing orbital character (Δ*E*_orb_) of the interaction varied to a lesser extent from −521.7 to −533.0 kcal mol^−1^, about −11 kcal mol^−1^, despite the marked decrease in the bent angle from 0Ni to 3Ni (116.2° to 70.3°), thus retaining a similar coordination environment. The electrostatic character of the interaction (Δ*E*_elstat_) varied from −668.2 to −649.6 kcal mol^−1^, denoting a decreasing variation of ∼19 kcal mol^−1^. The London interaction character (Δ*E*_disp_) remained similar along with the series from 0Ni to 3Ni, with a variation of <0.3 kcal mol^−1^. The Pauli repulsion (Δ*E*_Pauli_) varied by 11.4 kcal mol^−1^ along with the group, showing a more sterically demanding coordination site in the most bent structure, namely 3Ni. From these contributions, the energy difference associated with the nickel coordination was primarily due to a slight increase in the Pauli repulsion and a decrease in the stabilizing electrostatic character, which overcame the slightly more stabilizing orbital interaction variation, resulting in an overall destabilization as the dibenzoporphyrin(2.1.2.1) core bends.

**Fig. 3:**
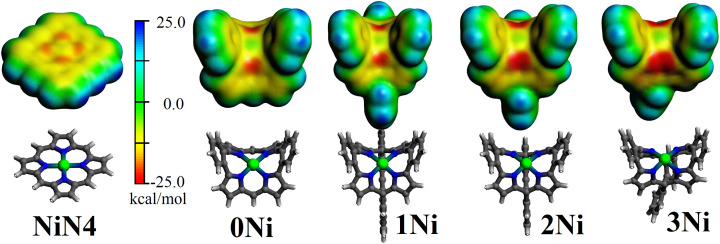
Electrostatic potential at the van der Waals surface (isosurface = 0.001 a.u.) for the 0Ni, 1Ni, 2Ni and 3Ni series. In addition, the unsubstituted nickel porphyrin (NiN4) is included.

Furthermore, the electrostatic potential at the van der Waals surface (Fig. 3) is a valuable tool in locating reactive sites within the resulting molecular structure. This is given by the formation of regions displaying a maximum in the surface electrostatic potential (*V*_S,max_). These are noted as σ-holes in clusters^59,60^ and as π-holes in metalloporphyrins,^61^ accounting for the capabilities of Lewis acidic regions^62^ leading to reactivity at the metal site. For nickel porphyrin, the π-hole site perpendicular to the N4 molecular plane^39^ showed a slight negative region in the range of −10.2 kcal mol^−1^ for the unsubstituted nickel porphyrin (NiN4) and of −2.7 kcal mol^−1^ for the nickel tetra(4-bromo-2,6-difluorophenyl) porphyrin (Nippy).^61^ Such features are in line with the observed nucleophilic character in square planar d^8^ complexes (as stated previously^61,63,64^) and denoted as electrophilic–nucleophilic dualism at such π-hole sites.^65^ For 0Ni, the electrostatic potential above the nickel metal center was −9.5 kcal mol^−1^, which increased to −10.3 kcal mol^−1^ in 1Ni. Upon reducing the angle between both *meso*-carbons and the N4-centroid to 116.2° in 2Ni and to 70.3° in 3Ni, the respective electrostatic potential amounted to −11.1 and −11.2 kcal mol^−1^.

To evaluate the characteristics of the π-hole site, we calculated the fluoride ion affinity (FIA) introduced by Bartlett.^66,67^ This is obtained as the negative of the enthalpy of the reaction of the π-hole with a fluoride ion in the gas phase, FIA^direct^ = −Δ*H*.^68^ Such an approach serves to classify the strength of the interaction in comparison with prototypical Lewis acids, provided by BF_3_, B(CF_3_)_3_, and B(C_6_F_5_)_3_ species.^69–72^

The calculated FIA for BF_3_ was 349.7 kJ mol^−1^, increasing to 441.1 kJ mol^−1^ for B(C_6_F_5_)_3_, and to 554.7 kJ mol^−1^ for B(CF_3_)_3_, which are in line with the reported values of 342, 452 kJ mol^−1^ and 556 kJ mol^−1^, respectively.^66,73^ For the planar nickel porphyrin (NiN_4_C_20_H_14_), the calculated FIA was 73.2 kJ mol^−1^, denoting weaker Lewis acid capabilities in comparison with BF_3_, B(CF_3_)_3_ and B(C_6_F_5_)_3_ species. Interestingly, in the unsubstituted nickel dibenzoporphyrin(2.1.2.1) (0Ni), the calculated FIA increased to 136.1 kJ mol^−1^, serving as a non-planar coordinating backbone modifying the characteristics of related planar metalloporphyrins. Upon inclusion of the butadiyne-bridge in the nickel dibenzoporphyrin(2.1.2.1) framework (1Ni), the calculated FIA increased further to 140.9, varying to 139.1 in 2Ni, and to 149.7 kJ mol^−1^ for 3Ni. These data suggested a mild Lewis acid capability for the resulting bent nickel dibenzoporphyrin(2.1.2.1).

**Fig. 4:**
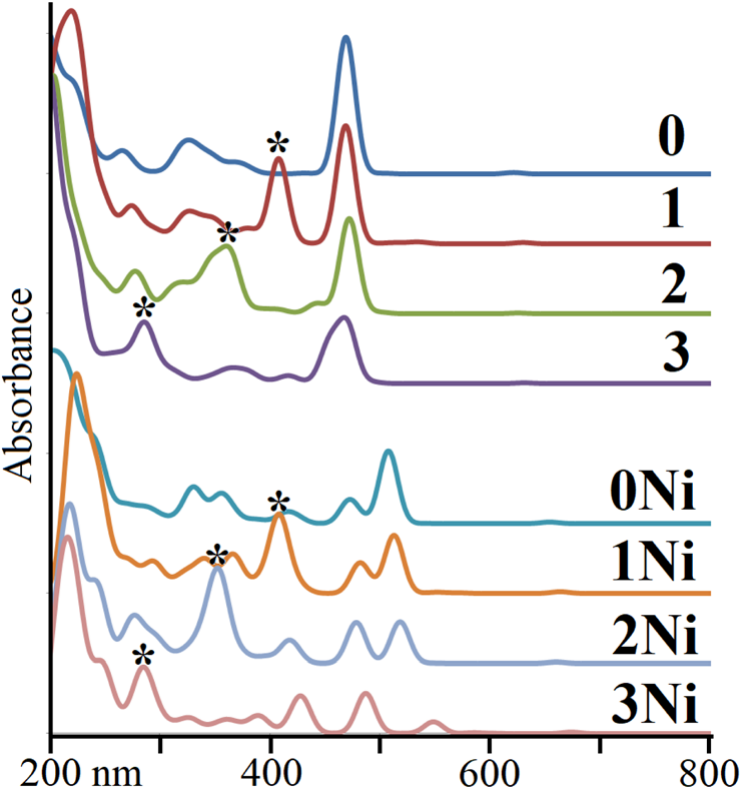
Calculated UV–Vis patterns for 0 to 3, and 0Ni to 3Ni series. The secondary peak is denoted by *.

We wished to evaluate the impact of shortening the connecting bridge from the studied series on the optical absorption pattern. Hence, TD-DFT calculations were carried out. For 1 and 1Ni, the first main absorption peak appeared at 431 and 534 nm (noted as Por and PorNi, respectively, in^40^), denoting a broad shape owing to the overlap of different signals. The calculated absorption bands appeared at 468 and 512 nm for 1 and 1Ni, respectively, within the method's error. Additionally, for 1 and 1Ni, smaller secondary bands were observed at 407 and 481 nm, respectively, which were expected to remain overlapped in the experimental characterization. In the 0 to 3 series, the first peak remained at a similar energy (468, 468, 471, and 467 nm), of π → π*-dibenzoporphyrin(2.1.2.1) character, whereas the secondary peak appearing in 1 to 3 varied as 407, 366 and 359 owing to a π-dibenzoporphyrin(2.1.2.1) →π*-bridge character. From 0Ni to 3Ni, the first peak varied as 507, 512, 517, and 486 nm, respectively, retaining a π → π*-dibenzoporphyrin(2.1.2.1) character, whereas the secondary peak varied as 407, 351, and 284 nm, of π-dibenzoporphyrin(2.1.2.1) →π*-bridge character. Hence, the secondary peak was affected more by the involved bridge motif (Fig. 4).

**Fig. 5:**
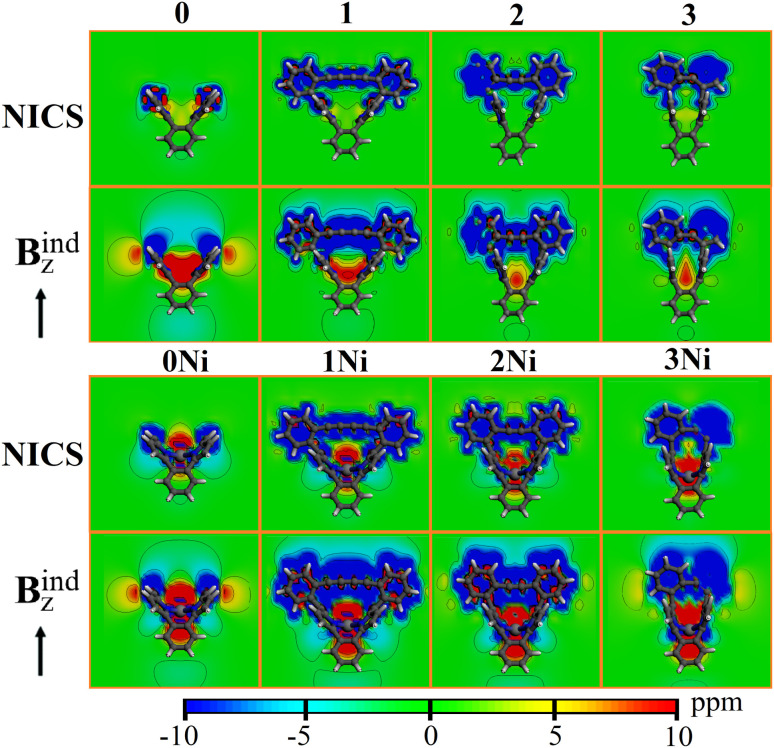
Contour plots of NICS and **B**_*z*_^ext^ for the studied species.

Finally, to gain insights into the effect of introducing the different rings in the studied species, the induced magnetic field was evaluated.^52,74^ This evaluation was in terms of NICS accounting for the orientationally averaged behavior owing to the constant molecular tumbling in solution, and under a specific orientation perpendicular to the N4-ring plane provided by **B**_*z*_^ind^. According to the NICS contour plots (Fig. 5), for 0, a central deshielding region (∼5.0 ppm) was located at the N4-ring, which decreased for 1 and 2, and increased to ∼5.0 ppm at 3. For **B**_*z*_^ind^, the central deshielding region was enhanced with values > 10.0 ppm, which was retained at the N4 bent from 1 to 3 (which faced the shielding contribution from the bridging motif). For the nickel counterparts, the central deshielding region was enhanced (as seen from NICS contour plots), facing the shielding region from the bridge, denoting that such a deshielding region was contained within the bent region. For **B**_*z*_^ind^, such observations were enhanced, indicating an increase in the deshielding region contained within the N4-cavity.

## Conclusions

Variation in the structural features in porphyrin- and metalloporphyrin-like species based on the bridged dibenzoporphyrin(2.1.2.1) motif was studied. The flexibility of the N4-core could be bent from 122.4° to 103.2°, and involved a strain energy of <3.4 kcal mol^−1^, and of 9.1 kcal mol^−1^ from 116.0° to 97.3° upon incorporation of the central nickel atom. Hence, the dibenzoporphyrin(2.1.2.1) core could be useful for further exploration of non-planar N4-motifs towards achieving tuned capabilities. Structural control of the bent angle could modify the HOMO–LUMO gap, and the optical absorption profiles could be determined. An increase in the Lewis acid capabilities of the resulting π-hole site perpendicular to the N4 molecular plane, in comparison with the pristine nickel porphyrin ring, was evaluated *via* FIA. Induced magnetic field analysis revealed a deshielding region to be retained within the bent cavity from N4- and NiN4-motifs in the bridged dibenzoporphyrin(2.1.2.1) species. These observations suggest controlled bending of N4 to be a useful synthetic strategy to tune the capabilities of porphyrin- and metalloporphyrin-like species.

## Conflicts of interest

There are no conflicts to declare.

## Supplementary Material

RA-015-D5RA07496G-s001

## Data Availability

The data supporting the conclusions reached from our study have been included as part of the supplementary information (SI). Supplementary information is available. See DOI: https://doi.org/10.1039/d5ra07496g.
