# Supplementary material for: Impact on increasing the bent angle in nickel dibenzoporphyrin(2.1.2.1) bows. Evaluation of structural and molecular features from computations
Source: RSC Adv. 2025 Oct 31;15(49):42229–37. doi: 10.1039/d5ra07496g (PMC12577750; doi:10.1039/d5ra07496g)
Supplement: RA-015-D5RA07496G-s001 [file RA-015-D5RA07496G-s001.pdf]

Supplementary Information (SI) for RSC Advances.
This journal is © The Royal Society of Chemistry 2025

Supporting information for:

Impact in Increasing the Bent Angle in Nickel Porphyrin(2.1.2.1) Bows. Evaluation of Structural and Molecular Features From Computations

Margot Paco-Chipana,[a] Alvaro Muñoz-Castro[b]*

[a]Estudiante de Doctorado en Biología Computacional, Facultad de Ingeniería, Universidad San Sebastián, Santiago, Chile.

[b]Facultad de Ingeniería, Universidad San Sebastián, Santiago, Chile.

Content

Figure S1. Isosurface for frontier orbitals of the studied species. Page S2

Table S1. Coordinates for the studied species in MultiXYZ format. Page S3

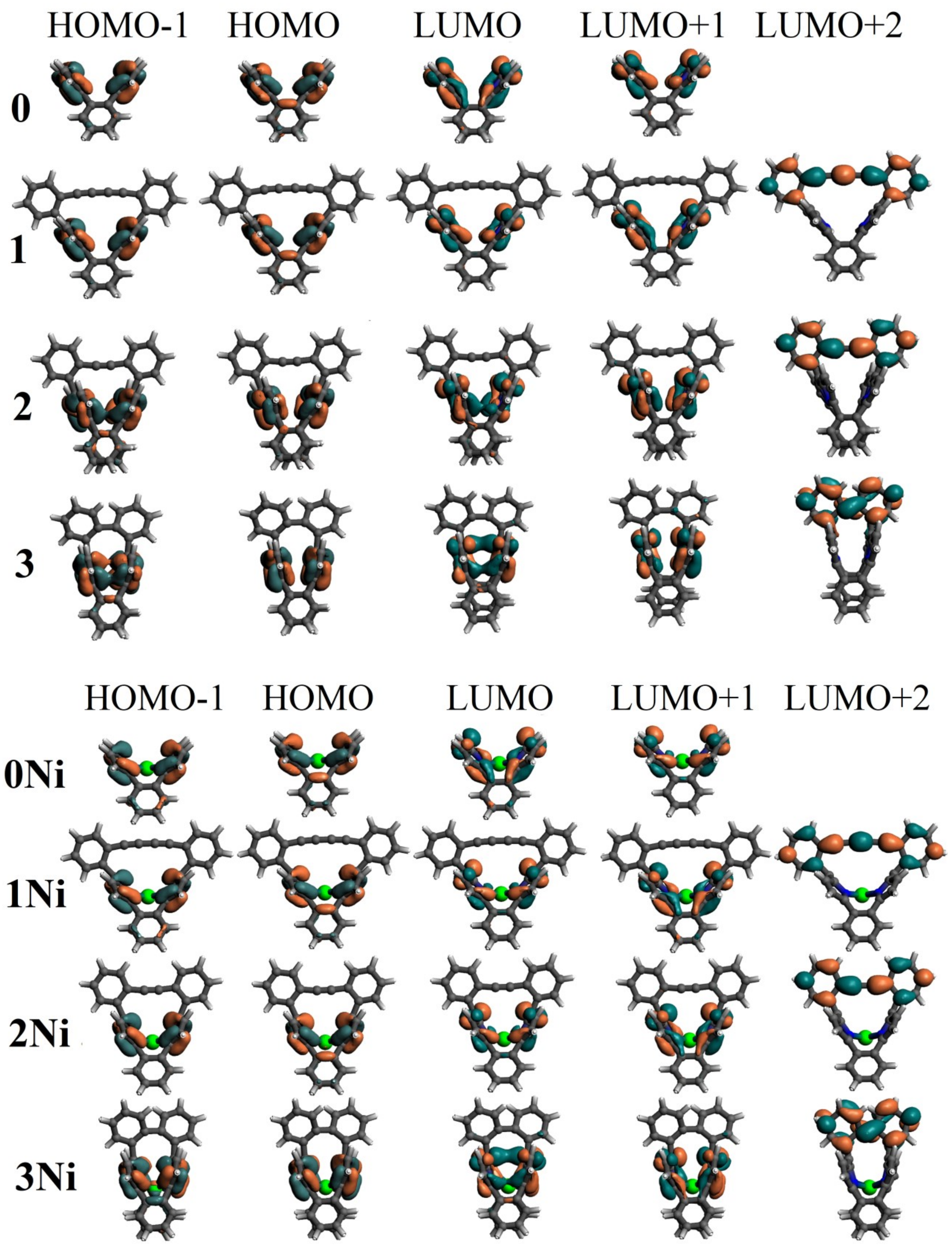

Figure S1. Isosurface for frontier orbitals of the studied species.

Table S1. Coordinates for the studied species in MultiXYZ format. Values in Angstrom (Å).

```
54
0
N        1.550200000      1.537200000      0.518000000
H        0.807300000      0.943400000      0.141000000
N       -1.550200000     -1.537200000      0.518000000
H       -0.807300000     -0.943400000      0.141000000
N       -1.337500000      1.447200000      0.469500000
N        1.337500000     -1.447200000      0.469500000
C        1.320400000      2.461300000      1.518500000
C        1.177400000     -2.333000000      1.547600000
C       -2.644900000      1.445000000      0.210800000
C       -1.177400000      2.333000000      1.547600000
C        0.043200000      2.773000000      2.019000000
C        3.296600000     -0.704100000     -0.889600000
C        2.857600000      1.553000000      0.149200000
C       -2.461900000      2.847700000      1.967200000
H       -2.629600000      3.546700000      2.781700000
C       -1.320400000     -2.461300000      1.518500000
C        2.562200000      3.076300000      1.798900000
H        2.718900000      3.846700000      2.546300000
C        3.424500000      0.704100000     -0.899900000
C       -0.043200000     -2.773000000      2.019000000
C       -3.424500000     -0.704100000     -0.899900000
C       -3.296600000      0.704100000     -0.889600000
C       -2.857600000     -1.553000000      0.149200000
C        3.938400000     -1.447600000     -1.893100000
H        3.849000000     -2.534100000     -1.873700000
C        2.644900000     -1.445000000      0.210800000
C        3.510900000      2.526400000      0.933200000
H        4.568500000      2.757400000      0.876900000
C        4.808300000      0.564300000     -2.900600000
H        5.387100000      1.062700000     -3.677600000
C        4.191900000      1.317200000     -1.905600000
H        4.286500000      2.402900000     -1.896700000
C        4.675900000     -0.826000000     -2.897900000
H        5.150100000     -1.425100000     -3.675800000
C       -2.562200000     -3.076300000      1.798900000
H       -2.718900000     -3.846700000      2.546300000
C       -3.386400000      2.306500000      1.113400000
H       -4.460600000      2.463400000      1.094300000
C        2.461900000     -2.847700000      1.967200000
H        2.629600000     -3.546700000      2.781700000
C       -4.191900000     -1.317200000     -1.905600000
H       -4.286500000     -2.402900000     -1.896700000
C       -3.510900000     -2.526400000      0.933200000
H       -4.568500000     -2.757400000      0.876900000
C        3.386400000     -2.306500000      1.113400000
H        4.460600000     -2.463400000      1.094300000
C       -3.938400000      1.447600000     -1.893100000
H       -3.849000000      2.534100000     -1.873700000
C       -4.808300000     -0.564300000     -2.900600000
H       -5.387100000     -1.062700000     -3.677600000
C       -4.675900000      0.826000000     -2.897900000
H       -5.150100000      1.425100000     -3.675800000
H        0.022200000      3.507900000      2.825000000
H       -0.022200000     -3.507900000      2.825000000
76
1
```

N	1.495900000	1.581700000	-0.810500000
H	0.694800000	1.069800000	-1.207400000
N	-1.495900000	-1.581700000	-0.810500000
H	-0.694800000	-1.069800000	-1.207400000
N	-1.278700000	1.511500000	-0.874800000
N	1.278700000	-1.511500000	-0.874800000
C	0.015300000	0.672500000	3.462500000
C	0.029000000	3.793800000	1.871500000
C	1.313700000	2.487100000	0.216900000
C	0.044800000	4.198200000	4.277500000
H	0.052500000	3.802500000	5.293200000
C	1.167600000	-2.392700000	0.213200000
C	0.038000000	3.290900000	3.198000000
C	0.034400000	1.894900000	3.398800000
C	-2.580100000	1.457300000	-1.152900000
C	-1.167600000	2.392700000	0.213200000
C	0.042600000	2.836700000	0.727000000
C	0.037600000	5.568500000	4.044100000
H	0.039900000	6.260600000	4.886800000
C	-0.015300000	-0.672500000	3.462500000
C	3.184400000	-0.702600000	-2.271800000
C	2.802600000	1.536600000	-1.174700000
C	-2.476800000	2.851700000	0.618900000
H	-2.678300000	3.530700000	1.442300000
C	-1.313700000	-2.487100000	0.216900000
C	2.586900000	3.020800000	0.524500000
H	2.776700000	3.754600000	1.300700000
C	0.017900000	5.171500000	1.658500000
H	0.007000000	5.540300000	0.632200000
C	3.326800000	0.702600000	-2.259900000
C	-0.042600000	-2.836700000	0.727000000
C	-3.326800000	-0.702600000	-2.259900000
C	-0.038000000	-3.290900000	3.198000000
C	-3.184400000	0.702600000	-2.271800000
C	-2.802600000	-1.536600000	-1.174700000
C	3.773600000	-1.431500000	-3.316300000
H	3.669300000	-2.517400000	-3.310700000
C	2.580100000	-1.457300000	-1.152900000
C	-0.034400000	-1.894900000	3.398800000
C	3.507700000	2.442100000	-0.352500000
H	4.579600000	2.609200000	-0.398400000
C	-0.029000000	-3.793800000	1.871500000
C	-0.044800000	-4.198200000	4.277500000
H	-0.052500000	-3.802500000	5.293200000
C	4.641600000	0.588900000	-4.307400000
H	5.205900000	1.094900000	-5.092000000
C	4.070700000	1.327500000	-3.275700000
H	4.179900000	2.411800000	-3.245000000
C	4.483500000	-0.797300000	-4.333200000
H	4.924000000	-1.387000000	-5.138000000
C	0.019900000	6.059200000	2.736600000
H	0.009300000	7.134400000	2.553300000
C	-2.586900000	-3.020800000	0.524500000
H	-2.776700000	-3.754600000	1.300700000
C	-3.367000000	2.278400000	-0.252000000
H	-4.448800000	2.384300000	-0.283000000
C	2.476800000	-2.851700000	0.618900000
H	2.678300000	-3.530700000	1.442300000
C	-4.070700000	-1.327500000	-3.275700000
H	-4.179900000	-2.411800000	-3.245000000
C	-3.507700000	-2.442100000	-0.352500000

H	-4.579600000	-2.609200000	-0.398400000
C	3.367000000	-2.278400000	-0.252000000
H	4.448800000	-2.384300000	-0.283000000
C	-3.773600000	1.431500000	-3.316300000
H	-3.669300000	2.517400000	-3.310700000
C	-0.037600000	-5.568500000	4.044100000
H	-0.039900000	-6.260600000	4.886800000
C	-0.017900000	-5.171500000	1.658500000
H	-0.007000000	-5.540300000	0.632200000
C	-4.641600000	-0.588900000	-4.307400000
H	-5.205900000	-1.094900000	-5.092000000
C	-4.483500000	0.797300000	-4.333200000
H	-4.924000000	1.387000000	-5.138000000
C	-0.019900000	-6.059200000	2.736600000
H	-0.009300000	-7.134400000	2.553300000
74			
2			
N	1.677000000	1.608200000	-1.022800000
H	0.822600000	1.261200000	-1.494800000
N	-1.677000000	-1.608200000	-1.022800000
H	-0.822600000	-1.261200000	-1.494800000
N	-1.004200000	1.520600000	-1.074400000
N	1.004200000	-1.520600000	-1.074400000
C	0.299600000	2.885100000	2.226000000
C	1.556400000	2.259600000	0.184100000
C	0.190900000	2.561700000	4.636300000
H	0.097400000	1.886900000	5.485600000
C	0.900800000	-1.969600000	0.255900000
C	0.178600000	2.011800000	3.335100000
C	0.055700000	0.606700000	3.192200000
C	-2.281000000	1.201100000	-1.249100000
C	-0.900800000	1.969600000	0.255900000
C	0.299100000	2.359700000	0.835600000
C	0.322100000	3.930400000	4.833000000
H	0.328700000	4.333000000	5.845400000
C	2.852300000	-0.666800000	-2.506200000
C	2.977100000	1.531000000	-1.402600000
C	-2.199000000	1.921900000	0.889600000
H	-2.417200000	2.214800000	1.911400000
C	-1.556400000	-2.259600000	0.184100000
C	2.861500000	2.644100000	0.570400000
H	3.114700000	3.163600000	1.488200000
C	0.435600000	4.258600000	2.443600000
H	0.526400000	4.916000000	1.579800000
C	3.330500000	0.666800000	-2.533800000
C	-0.299100000	-2.359700000	0.835600000
C	-3.330500000	-0.666800000	-2.533800000
C	-0.178600000	-2.011800000	3.335100000
C	-2.852300000	0.666800000	-2.506200000
C	-2.977100000	-1.531000000	-1.402600000
C	3.048500000	-1.485200000	-3.622200000
H	2.672800000	-2.507400000	-3.597400000
C	2.281000000	-1.201100000	-1.249100000
C	-0.055700000	-0.606700000	3.192200000
C	3.742600000	2.207900000	-0.430600000
H	4.823200000	2.296100000	-0.437900000
C	-0.299600000	-2.885100000	2.226000000
C	-0.190900000	-2.561700000	4.636300000
H	-0.097400000	-1.886900000	5.485600000
C	4.264800000	0.285900000	-4.736100000
H	4.821300000	0.651500000	-5.598800000

C	4.050600000	1.124600000	-3.643000000
H	4.410600000	2.153000000	-3.655300000
C	3.745400000	-1.010000000	-4.734600000
H	3.894800000	-1.658200000	-5.597400000
C	0.445200000	4.785200000	3.734800000
H	0.546500000	5.859600000	3.882600000
C	-2.861500000	-2.644100000	0.570400000
H	-3.114700000	-3.163600000	1.488200000
C	-3.065700000	1.422100000	-0.047600000
H	-4.128500000	1.230500000	0.055100000
C	2.199000000	-1.921900000	0.889600000
H	2.417200000	-2.214800000	1.911400000
C	-4.050600000	-1.124600000	-3.643000000
H	-4.410600000	-2.153000000	-3.655300000
C	-3.742600000	-2.207900000	-0.430600000
H	-4.823200000	-2.296100000	-0.437900000
C	3.065700000	-1.422100000	-0.047600000
H	4.128500000	-1.230500000	0.055100000
C	-3.048500000	1.485200000	-3.622200000
H	-2.672800000	2.507400000	-3.597400000
C	-0.322100000	-3.930400000	4.833000000
H	-0.328700000	-4.333000000	5.845400000
C	-0.435600000	-4.258600000	2.443600000
H	-0.526400000	-4.916000000	1.579800000
C	-4.264800000	-0.285900000	-4.736100000
H	-4.821300000	-0.651500000	-5.598800000
C	-3.745400000	1.010000000	-4.734600000
H	-3.894800000	1.658200000	-5.597400000
C	-0.445200000	-4.785200000	3.734800000
H	-0.546500000	-5.859600000	3.882600000
72			
3			
N	1.588100000	1.422300000	-1.008600000
N	-1.572000000	-1.449500000	-0.998500000
N	-1.099300000	1.388300000	-1.047900000
N	1.116500000	-1.437300000	-1.017500000
C	0.099600000	1.803900000	2.476900000
C	1.504100000	1.724800000	0.354900000
C	-0.769300000	1.181900000	4.629100000
H	-1.094800000	0.410200000	5.323700000
C	0.970600000	-1.592400000	0.352800000
C	-0.185800000	0.765100000	3.407100000
C	-2.413000000	1.263200000	-1.376200000
C	-0.965200000	1.579500000	0.318200000
C	0.277500000	1.658100000	1.001600000
C	-0.944100000	2.513400000	4.987300000
H	-1.404400000	2.758500000	5.942800000
C	2.950800000	-0.691800000	-2.584500000
C	2.881900000	1.415500000	-1.314300000
C	-2.273500000	1.654500000	0.850900000
H	-2.514100000	1.811200000	1.895000000
C	-1.499800000	-1.733400000	0.370300000
C	2.826100000	1.977600000	0.875300000
H	3.066000000	2.243500000	1.900500000
C	-0.026800000	3.147400000	2.875500000
H	0.225200000	3.912400000	2.141100000
C	3.288700000	0.691800000	-2.539300000
C	-0.277500000	-1.658100000	1.026800000
C	-3.259500000	-0.734500000	-2.554300000
C	0.143100000	-0.717700000	3.423200000
C	-2.929300000	0.649100000	-2.620900000

C	-2.862900000	-1.436500000	-1.313300000
C	3.235300000	-1.436600000	-3.730000000
H	2.984000000	-2.497100000	-3.753100000
C	2.429900000	-1.278600000	-1.330300000
C	3.694800000	1.817700000	-0.182400000
H	4.777500000	1.904400000	-0.173600000
C	-0.114100000	-1.773900000	2.504200000
C	0.707500000	-1.111600000	4.662800000
H	1.012300000	-0.328400000	5.353200000
C	4.223000000	0.512700000	-4.768400000
H	4.720500000	0.978900000	-5.618700000
C	3.939400000	1.271200000	-3.630000000
H	4.199000000	2.329100000	-3.593900000
C	3.862100000	-0.834000000	-4.824200000
H	4.079100000	-1.422100000	-5.715700000
C	-0.514400000	3.516100000	4.123200000
H	-0.602900000	4.568200000	4.390800000
C	-2.827600000	-1.964000000	0.885200000
H	-3.079900000	-2.202500000	1.913900000
C	-3.167600000	1.473900000	-0.204800000
H	-4.249700000	1.425100000	-0.152900000
C	2.271700000	-1.615400000	0.904600000
H	2.501600000	-1.732200000	1.955000000
C	-3.903400000	-1.336400000	-3.637600000
H	-4.157400000	-2.395100000	-3.583800000
C	-3.687500000	-1.812100000	-0.180600000
H	-4.771200000	-1.884400000	-0.174700000
C	3.174100000	-1.436000000	-0.144300000
H	4.250700000	-1.349400000	-0.078300000
C	-3.221400000	1.374400000	-3.777000000
H	-2.976900000	2.435900000	-3.816500000
C	0.892000000	-2.436200000	5.043200000
H	1.338600000	-2.661800000	6.011300000
C	0.019400000	-3.108700000	2.925500000
H	-0.208300000	-3.886600000	2.197800000
C	-4.190600000	-0.598500000	-4.788200000
H	-4.683000000	-1.080800000	-5.632700000
C	-3.841100000	0.750000000	-4.862800000
H	-4.061700000	1.322500000	-5.763500000
C	0.490700000	-3.455500000	4.185700000
H	0.589000000	-4.504000000	4.466100000
H	0.262800000	-1.295400000	-1.580400000
H	-0.243400000	1.236300000	-1.602000000
53			
0Ni			
Ni	0.000000000	0.000000000	0.628300000
N	-1.340600000	-1.349900000	0.785100000
N	-1.340600000	1.349900000	0.785100000
N	1.340600000	-1.349900000	0.785100000
N	1.340600000	1.349900000	0.785100000
C	1.229100000	-2.308400000	1.795900000
C	2.466600000	-3.011600000	1.901800000
H	2.677600000	-3.814600000	2.602200000
C	3.290500000	-2.519700000	0.906500000
H	4.299900000	-2.836400000	0.663400000
C	2.569600000	-1.482000000	0.239400000
C	3.068600000	-0.705800000	-0.894300000
C	3.656600000	-1.389700000	-1.971300000
H	3.656700000	-2.479600000	-1.963000000
C	4.219300000	-0.698900000	-3.040800000
H	4.656100000	-1.249100000	-3.874100000

C	4.219300000	0.698900000	-3.040800000
H	4.656100000	1.249100000	-3.874100000
C	3.656600000	1.389700000	-1.971300000
H	3.656700000	2.479600000	-1.963000000
C	3.068600000	0.705800000	-0.894300000
C	2.569600000	1.482000000	0.239400000
C	3.290500000	2.519700000	0.906500000
H	4.299900000	2.836400000	0.663400000
C	2.466600000	3.011600000	1.901800000
H	2.677600000	3.814600000	2.602200000
C	1.229100000	2.308400000	1.795900000
C	0.000000000	2.702100000	2.315900000
C	0.000000000	-2.702100000	2.315900000
C	-1.229100000	-2.308400000	1.795900000
C	-2.466600000	-3.011600000	1.901800000
H	-2.677600000	-3.814600000	2.602200000
C	-3.290500000	-2.519700000	0.906500000
H	-4.299900000	-2.836400000	0.663400000
C	-2.569600000	-1.482000000	0.239400000
C	-3.068600000	-0.705800000	-0.894300000
C	-3.656600000	-1.389700000	-1.971300000
H	-3.656700000	-2.479600000	-1.963000000
C	-4.219300000	-0.698900000	-3.040800000
H	-4.656100000	-1.249100000	-3.874100000
C	-4.219300000	0.698900000	-3.040800000
H	-4.656100000	1.249100000	-3.874100000
C	-3.656600000	1.389700000	-1.971300000
H	-3.656700000	2.479600000	-1.963000000
C	-3.068600000	0.705800000	-0.894300000
C	-2.569600000	1.482000000	0.239400000
C	-3.290500000	2.519700000	0.906500000
H	-4.299900000	2.836400000	0.663400000
C	-2.466600000	3.011600000	1.901800000
H	-2.677600000	3.814600000	2.602200000
C	-1.229100000	2.308400000	1.795900000
H	0.000000000	3.492100000	3.068400000
H	0.000000000	-3.492100000	3.068400000

75
1Ni

Ni	-0.002181000	0.015345000	0.003945000
N	-0.699754000	1.756749000	-0.343976000
N	1.778797000	0.692143000	-0.073858000
N	-1.761670000	-0.685746000	-0.220510000
N	0.718278000	-1.751235000	0.036255000
C	-2.527646000	-0.291989000	-1.320389000
C	-3.650443000	-1.166136000	-1.437617000
H	-4.415044000	-1.098440000	-2.203698000
C	-3.594188000	-2.030768000	-0.361194000
H	-4.291249000	-2.820339000	-0.099642000
C	-2.405376000	-1.719358000	0.366578000
C	-1.970249000	-2.392655000	1.588062000
C	-2.900563000	-2.577190000	2.623932000
H	-3.893988000	-2.142799000	2.514967000
C	-2.566386000	-3.289143000	3.771402000
H	-3.298811000	-3.408342000	4.569374000
C	-1.292728000	-3.849369000	3.893518000
H	-1.022026000	-4.409979000	4.787711000
C	-0.366241000	-3.688417000	2.868053000
H	0.629052000	-4.123812000	2.951690000
C	-0.682685000	-2.954806000	1.713062000
C	0.300066000	-2.889544000	0.633616000

C	0.994204000	-4.011228000	0.086490000
H	0.843118000	-5.041389000	0.391526000
C	1.844021000	-3.527364000	-0.889785000
H	2.537080000	-4.083918000	-1.510886000
C	1.706600000	-2.106577000	-0.885199000
C	2.601354000	-1.180839000	-1.430397000
C	3.611765000	-1.657770000	-2.414795000
C	4.833982000	-2.195154000	-2.015407000
H	5.051323000	-2.270016000	-0.950471000
C	5.763614000	-2.626737000	-2.963097000
H	6.715661000	-3.044068000	-2.636881000
C	5.472316000	-2.523076000	-4.325521000
H	6.197013000	-2.860193000	-5.065755000
C	4.257700000	-1.991422000	-4.743473000
H	4.019480000	-1.906738000	-5.802394000
C	3.310967000	-1.551644000	-3.797004000
C	2.065183000	-1.007678000	-4.173716000
C	0.955578000	-0.527348000	-4.365924000
C	-0.278917000	-0.011391000	-4.498656000
C	-1.406999000	0.462267000	-4.541926000
C	-2.702700000	1.009091000	-4.429089000
C	-3.495238000	1.307603000	-5.555679000
H	-3.091413000	1.114069000	-6.548172000
C	-4.770595000	1.837179000	-5.395013000
H	-5.375107000	2.064293000	-6.272704000
C	-5.277106000	2.077440000	-4.115270000
H	-6.276783000	2.492047000	-3.990660000
C	-4.501001000	1.788253000	-2.991067000
H	-4.884523000	1.973914000	-1.988347000
C	-3.220008000	1.258085000	-3.131430000
C	-2.365019000	0.943521000	-1.953437000
C	-1.544822000	1.952059000	-1.438951000
C	-1.669957000	3.353502000	-1.681255000
H	-2.264262000	3.798159000	-2.471823000
C	-0.962711000	3.993653000	-0.681404000
H	-0.849245000	5.060575000	-0.519595000
C	-0.361176000	2.979162000	0.123915000
C	0.461148000	3.223944000	1.306659000
C	-0.007738000	4.122550000	2.279206000
H	-1.001392000	4.551965000	2.154757000
C	0.767306000	4.451098000	3.386994000
H	0.377735000	5.137797000	4.138167000
C	2.042196000	3.898081000	3.530336000
H	2.657115000	4.149343000	4.394271000
C	2.528103000	3.023467000	2.563479000
H	3.524132000	2.592273000	2.661889000
C	1.748883000	2.666627000	1.450858000
C	2.343287000	1.815270000	0.422910000
C	3.622507000	2.025004000	-0.176343000
H	4.282068000	2.856473000	0.048042000
C	3.819507000	1.001101000	-1.082973000
H	4.682229000	0.823424000	-1.715668000
C	2.685119000	0.140363000	-0.982317000

73

2Ni

N	-1.333500000	-1.331800000	-0.923100000
N	1.339200000	1.334600000	-0.915900000
N	1.341500000	-1.331300000	-0.915100000
N	-1.334500000	1.335900000	-0.921100000
C	-0.006300000	-2.910400000	2.241600000
C	-1.229500000	-2.075100000	0.254300000

C	-0.010600000	-2.584300000	4.653800000
H	-0.008700000	-1.908000000	5.507500000
C	-1.228000000	2.077400000	0.257400000
C	-0.003900000	-2.028500000	3.354700000
C	0.002500000	-0.613700000	3.210400000
C	2.612400000	-1.441700000	-1.349900000
C	1.230900000	-2.073800000	0.262100000
C	-0.001100000	-2.323100000	0.878300000
C	-0.020300000	-3.962600000	4.842500000
H	-0.024800000	-4.368400000	5.854600000
C	-3.134200000	0.712000000	-2.514000000
C	-2.602200000	-1.439500000	-1.365700000
C	2.500500000	-2.652100000	0.558500000
H	2.713400000	-3.293000000	1.407500000
C	1.232000000	2.075100000	0.262300000
C	-2.501700000	-2.650900000	0.543900000
H	-2.720200000	-3.290600000	1.392300000
C	-0.015000000	-4.290700000	2.448300000
H	-0.016000000	-4.949600000	1.580300000
C	-3.129300000	-0.701500000	-2.518300000
C	0.001100000	2.323100000	0.881000000
C	3.138100000	0.706800000	-2.505700000
C	0.002900000	2.021100000	3.356300000
C	3.143900000	-0.706800000	-2.502000000
C	2.609200000	1.443000000	-1.353200000
C	-3.758800000	1.399500000	-3.563900000
H	-3.766700000	2.488600000	-3.546800000
C	-2.604300000	1.447100000	-1.360900000
C	0.002600000	0.606400000	3.210500000
C	-3.347300000	-2.290300000	-0.494700000
H	-4.389500000	-2.560300000	-0.630600000
C	0.000400000	2.906200000	2.246000000
C	0.005800000	2.572400000	4.656400000
H	0.008000000	1.893600000	5.507800000
C	-4.349100000	-0.689600000	-4.618600000
H	-4.815600000	-1.235800000	-5.437100000
C	-3.748900000	-1.386400000	-3.571800000
H	-3.750000000	-2.475600000	-3.561000000
C	-4.354700000	0.706200000	-4.614500000
H	-4.825800000	1.255200000	-5.429700000
C	-0.022500000	-4.821400000	3.740400000
H	-0.029500000	-5.901700000	3.886200000
C	2.503300000	2.650000000	0.557200000
H	2.718900000	3.288700000	1.406600000
C	3.352100000	-2.293000000	-0.474300000
H	4.394700000	-2.563900000	-0.603900000
C	-2.498300000	2.657000000	0.548200000
H	-2.714000000	3.297800000	1.396400000
C	3.755700000	1.392600000	-3.560400000
H	3.756300000	2.481900000	-3.549300000
C	3.352600000	2.289700000	-0.476300000
H	4.395600000	2.558600000	-0.607100000
C	-3.346200000	2.298600000	-0.487800000
H	-4.388300000	2.570100000	-0.620700000
C	3.767500000	-1.393600000	-3.552600000
H	3.776800000	-2.482800000	-3.534800000
C	0.005000000	3.949600000	4.849000000
H	0.006100000	4.351700000	5.862300000
C	-0.000900000	4.285500000	2.456600000
H	-0.002900000	4.946800000	1.590700000
C	4.352800000	0.696900000	-4.609500000

H	4.817400000	1.244100000	-5.429500000
C	4.359600000	-0.699200000	-4.605400000
H	4.829600000	-1.247200000	-5.421500000
C	0.001300000	4.811700000	3.750000000
H	0.000000000	5.891200000	3.898800000
Ni	0.003100000	0.001500000	-1.164400000
71			
3Ni			
N	1.356600000	1.257200000	-0.975300000
N	-1.368700000	-1.264600000	-0.964200000
N	-1.311300000	1.331100000	-1.078500000
N	1.298700000	-1.335400000	-1.083400000
C	-0.210600000	1.796300000	2.463000000
C	1.228200000	1.608200000	0.377100000
C	-0.901700000	0.999100000	4.619800000
H	-1.078800000	0.172200000	5.303900000
C	1.228400000	-1.756600000	0.249500000
C	-0.278500000	0.705500000	3.379500000
C	-2.592600000	1.443200000	-1.478400000
C	-1.233500000	1.752500000	0.253900000
C	-0.028200000	1.687100000	0.989100000
C	-1.288700000	2.274400000	5.013700000
H	-1.759700000	2.418200000	5.985900000
C	3.102400000	-0.706500000	-2.652100000
C	2.660900000	1.319800000	-1.297600000
C	-2.530300000	2.190900000	0.656600000
H	-2.789400000	2.592000000	1.629200000
C	-1.231900000	-1.605300000	0.390300000
C	2.520500000	1.932900000	0.880700000
H	2.732200000	2.274700000	1.888500000
C	-0.549100000	3.090300000	2.896600000
H	-0.440000000	3.901700000	2.177500000
C	3.173500000	0.706500000	-2.536200000
C	0.028200000	-1.687100000	0.993100000
C	-3.193700000	-0.712600000	-2.514300000
C	0.303800000	-0.701800000	3.378100000
C	-3.123400000	0.699900000	-2.635500000
C	-2.676300000	-1.318700000	-1.273800000
C	3.642000000	-1.332300000	-3.779100000
H	3.593000000	-2.418000000	-3.856400000
C	2.577700000	-1.447900000	-1.490800000
C	3.409700000	1.797800000	-0.178500000
H	4.479100000	1.980400000	-0.173300000
C	0.223600000	-1.795400000	2.465500000
C	0.945000000	-0.991600000	4.609800000
H	1.131700000	-0.162400000	5.288800000
C	4.305300000	0.813000000	-4.677400000
H	4.767800000	1.403600000	-5.467900000
C	3.786900000	1.449500000	-3.549100000
H	3.845500000	2.533200000	-3.450200000
C	4.230800000	-0.575800000	-4.793600000
H	4.634800000	-1.072600000	-5.675100000
C	-1.053300000	3.351800000	4.165100000
H	-1.300600000	4.370700000	4.460500000
C	-2.522500000	-1.912200000	0.908300000
H	-2.728500000	-2.242700000	1.921100000
C	-3.365500000	2.047100000	-0.442800000
H	-4.421400000	2.287400000	-0.508800000
C	2.527800000	-2.195600000	0.644200000
H	2.793200000	-2.595300000	1.615600000
C	-3.808000000	-1.460400000	-3.522800000

H	-3.864800000	-2.544100000	-3.420300000
C	-3.420200000	-1.777700000	-0.143600000
H	-4.491500000	-1.950700000	-0.127200000
C	3.356600000	-2.050900000	-0.459700000
H	4.412500000	-2.290800000	-0.531400000
C	-3.665700000	1.321600000	-3.763400000
H	-3.617600000	2.407500000	-3.845300000
C	1.338100000	-2.265800000	5.001400000
H	1.822700000	-2.407000000	5.966800000
C	0.567200000	-3.089000000	2.898200000
H	0.449900000	-3.902300000	2.182600000
C	-4.330900000	-0.828200000	-4.651600000
H	-4.796000000	-1.422100000	-5.438200000
C	-4.257900000	0.560700000	-4.772900000
H	-4.665800000	1.054200000	-5.654400000
C	1.089400000	-3.345700000	4.159800000
H	1.340100000	-4.363800000	4.455600000
Ni	-0.007100000	-0.003100000	-1.406200000
